# Unusual orbital lymphoma undetectable by magnetic resonance imaging: a case report

**DOI:** 10.1186/1752-1947-3-104

**Published:** 2009-11-03

**Authors:** Maria Tatsugawa, Hidetaka Noma, Tatsuya Mimura, Hideharu Funatsu

**Affiliations:** 1 Department of Ophthalmology, Hiroshima Prefectural Hospital, Hiroshima, Japan, 1-5-54, Ujinakanda, Minami-ku, Hiroshima 734-8530, Japan; 2 Department of Ophthalmology, Yachiyo Medical Center, Tokyo Women's Medical University, Tokyo, Japan, 477-96, Owada-shinden, Yachiyo, Chiba 276-8524, Japan; 3 Department of Ophthalmology, University of Tokyo Graduate School of Medicine, Tokyo, Japan, 7-3-1, Hongo, Bunkyo-ku, Tokyo 113-0033, Japan

## Abstract

**Introduction:**

We report the case of a patient with orbital malignant lymphoma that was not detected by imaging studies when she presented with impaired vision, which lead to her eventual loss of sight.

**Case presentation:**

A 71-year-old Japanese woman complained of deteriorating vision in her left eye. On examination, papilledema was detected, but magnetic resonance imaging only showed slight thickening and enhancement of the left optic nerve. A diagnosis of idiopathic optic neuritis was made and corticosteroid pulse therapy was administered. During the next four months, the patient received a total of four courses of corticosteroid pulse therapy, but she still suffered from bilateral loss of vision. A second magnetic resonance imaging procedure revealed tumors in both orbits and a biopsy showed diffuse large B-cell malignant lymphoma.

**Conclusion:**

The possibility of malignant lymphoma should be considered in patients with recurrent optic neuropathy despite administration of corticosteroid pulse therapy, even when there are no abnormalities on cerebrospinal fluid examination or magnetic resonance imaging.

## Introduction

Primary malignant lymphoma is relatively frequent among orbital tumors [1] and is usually not difficult to detect because it forms a mass lesion that causes changes such as proptosis [2]. Primary malignant lymphoma of the orbit can present with palpebral swelling, proptosis, tumor formation, diplopia, blepharoptosis, and ocular displacement [1], but few authors have reported orbital lymphoma presenting with optic neuropathy [2]. In patients with visual disorders, imaging generally reveals compression of the optic nerve by the tumor.

Here we report the case of a patient with orbital lymphoma that was not detected by imaging studies at the time when she developed the initial symptom of visual impairment, and which eventually lead to loss of sight. It is very rare for imaging studies to only show optic nerve swelling despite marked bilateral loss of vision, as occurred in this case.

## Case presentation

**Figure 1:**
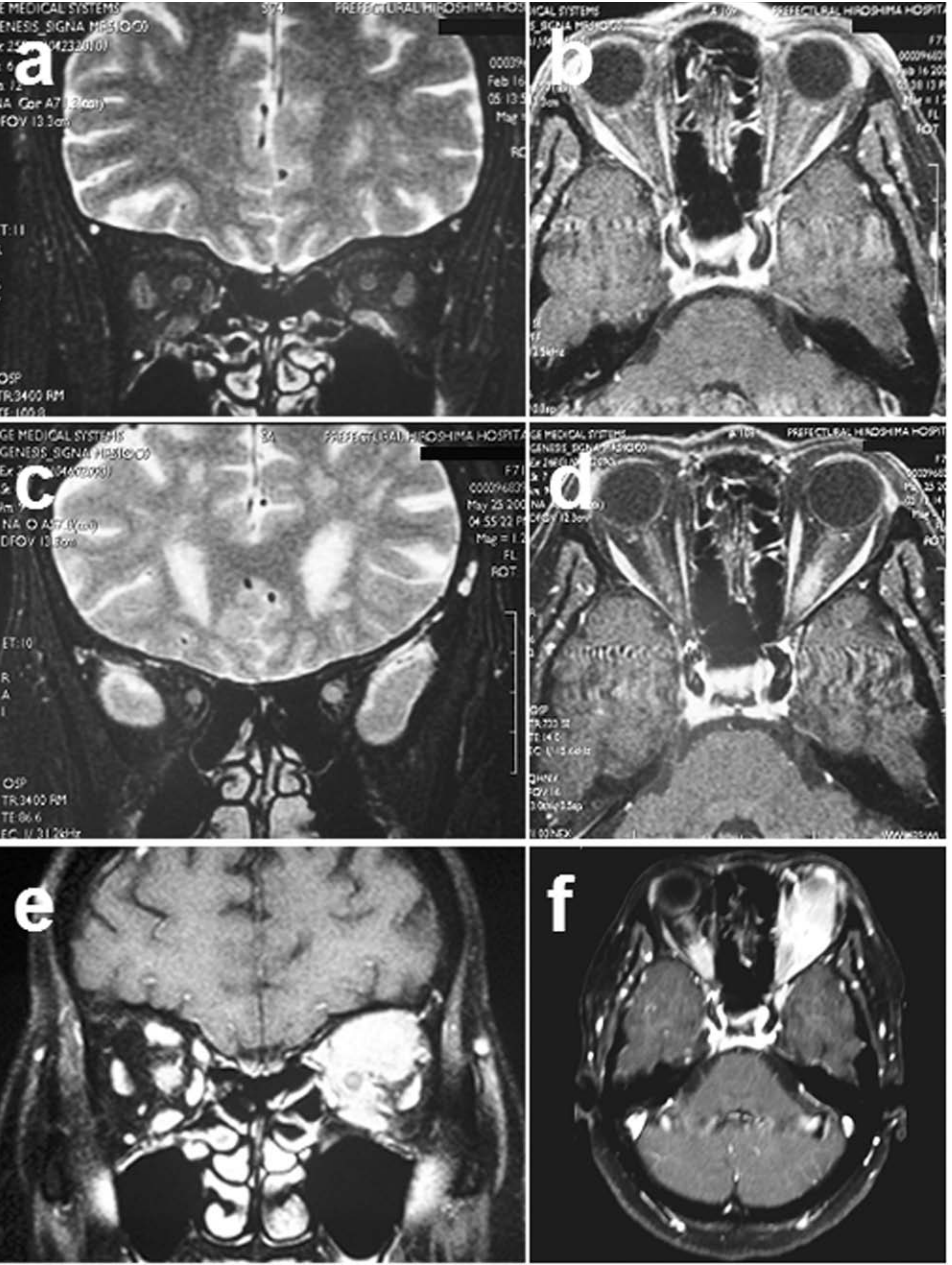
**Magnetic resonance images of the head and orbits**. (a) Initial coronal image (unenhanced T2-weighted) showing the left optic nerve to be thicker and more enhanced than the right optic nerve. (b) Transverse (enhanced T1-weighted) coronal image showing the left optic nerve to be thicker and more enhanced than the right optic nerve. (c) Coronal (enhanced T2-weighted) image obtained after loss of vision in both eyes, which shows that the right optic nerve has high signal intensity while the left optic nerve is swollen. (d) Transverse (enhanced T1-weighted) coronal image obtained after loss of vision in both eyes, which shows that the right optic nerve has high signal intensity while the left optic nerve is swollen. (e) Coronal (enhanced T1-weighted) image obtained six months after presentation displays bilateral orbital tumors. (f) Transverse (enhanced T1-weighted) coronal image obtained six months after presentation displays bilateral orbital tumors.

On the 2nd February 2005, a previously healthy 71-year-old Japanese woman consulted our hospital due to reduced vision in the left eye. A full ophthalmological examination revealed that her visual acuity was 20/20 in the right eye and 20/60 in the left eye. There was no relative afferent pupil defect (RAPD). On funduscopy, the left eye showed papilledema. Goldmann perimetry of the left eye showed mild central depression, enlargement of Mariotte's blind spot, and paracentral scotoma. Fluorescein angiography showed early hyperfluorescence of the disc. The results of hematology tests, renal and liver function tests, urine analysis, chest radiography, and computed tomography were all within normal limits. Magnetic resonance imaging (MRI) of the head and orbits showed that the left optic nerve was slightly thicker than the right optic nerve (Figure 1a, b).

A diagnosis of idiopathic optic neuritis was made and corticosteroid pulse therapy was started on February 3rd. After four days, her visual acuity was 20/20 oculus sinister (OS) and the swelling of the left optic disk had already resolved. The patient's response to corticosteroid therapy and her age suggested a diagnosis of autoimmune optic neuritis. On March 14th and April 12th, visual acuity was reduced to 20/200 OS. There was neither RAPD nor any abnormal ocular findings. Corticosteroid pulse therapy was started again because her vision had deteriorated after reduction of the steroid dose. However, visual acuity on the left side declined to no light perception by May 10th.

On May 20th, the right eye showed a decrease of acuity to 20/600 despite the absence of abnormal ocular findings. On May 24th, the patient was again started on corticosteroid pulse therapy. An MRI of the head and orbits revealed swelling of the left optic nerve (Figure 1c, d). Cerebrospinal fluid (CSF) examination did not show any increase in cell count, abnormal cells or any other findings. On June 3rd, hemorrhage was detected around the left optic disk. On June 10th, slight papilledema was observed on the patient's right eye. Visual acuity declined to no light perception on the right side by June 21st. On July 4th, she complained of left eye pain, and proptosis and subcutaneous hemorrhage were noted around the eyes which suggested thrombosis of tumor pressure.

**Figure 2:**
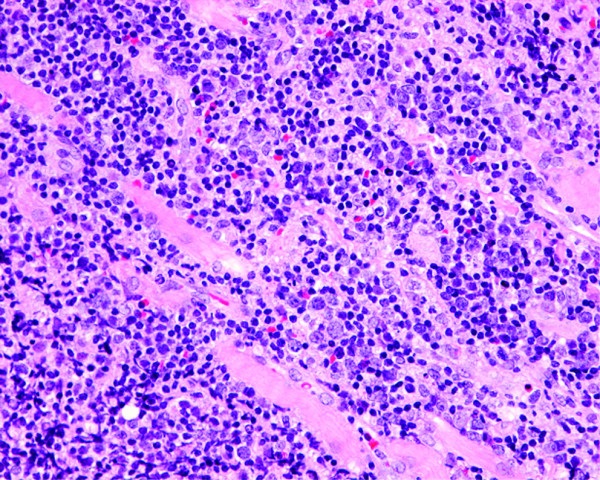
**The resected orbital tumor shows infiltration of the extraocular muscles**. Tumor biopsy was carried out six months after presentation. The tumor cells are large, polygonal, or round and the nuclei are folded, cleaved, or kidney-shaped. Mitoses and prominent nuclear abnormalities can be seen. The pathological diagnosis was diffuse large B cell lymphoma (hematoxylin-eosin, × 50).

On July 5th, an MRI revealed mass lesions in the patient's left and right orbits. Three days later, an orbital tumor biopsy was performed at the Department of Neurosurgery (Figure 1e, f). Pathological examination gave a diagnosis of diffuse large B-cell lymphoma (Figure 2). Imaging studies showed intraparenchymal optic nerve disease, thus suggesting the possibility of breakout into the surrounding orbital soft tissue because tumor cells were found in the soft tissue and fibroadipose tissue through orbital tumor biopsy.

Immunohistochemical studies showed that the tumor cells were negative for expression of T cell markers (CD3 and CD56) and for Ki-1 (CD30), which is a marker of Hodgkin's and Reed-Sternberg cells. The cells were also negative for cell marker p53 and Epstein-Barr virus-encoded small RNA. Instead, the atypical lymphocytes expressed the B cell markers CD20 and CD79a (data not shown).

On July 28th, the patient was referred to another hospital for chemotherapy and radiotherapy. The succeeding 42 months showed no evidence of local recurrence or systemic metastasis of the lymphoma.

## Discussion

The tumor found in our patient may have developed around her optic nerves and caused compression until the second course of corticosteroid pulse therapy was administered. Although visual acuity recovered during corticosteroid pulse therapy, it deteriorated again after the dose was decreased. These dose-dependent changes of the visual symptoms suggest that circulatory impairment related to the perivascular infiltration of tumor cells led to rapid loss of vision during the period before the tumor could be detected by MRI.

Almost all orbital lymphomas can be diagnosed from swelling of the optic nerve on imaging studies and from abnormal findings upon examination of the CSF [3-5]. However, there was no optic nerve swelling in our patient until after the third course of corticosteroid pulse therapy was administered. Therefore, orbital lymphoma can sometimes cause loss of sight without being detected by imaging.

## Conclusion

The possibility of orbital lymphoma should be considered in patients with an early recurrence of optic neuropathy after corticosteroid pulse therapy, even when there are no abnormal findings on CSF examination or MRI.

## Abbreviations

MRI: magnetic resonance imaging; OS: oculus sinister (Latin for "left eye"); RAPD: relative afferent pupil defect.

## Consent

Written informed consent was obtained from the patient for publication of this case report and any accompanying images. A copy of the written consent is available for review by the Editor-in-Chief of this journal.

## Competing interests

The authors declare that they have no competing interests.

## Authors' contributions

MT, HN, TM and HF analyzed and interpreted the patient's medical data. MT was a major contributor in writing the manuscript. All authors read and approved the final manuscript.
